# The Impact of Long-Term Parenteral Nutrition on Physical Development and Bone Mineralization in Children with Chronic Intestinal Failure

**DOI:** 10.3390/nu17040611

**Published:** 2025-02-07

**Authors:** Hanna Romanowska, Mikołaj Danko, Katarzyna Popińska, Joanna Żydak, Marta Sibilska, Joanna Wielopolska, Klaudia Bartoszewicz, Anna Borkowska, Mieczysław Walczak, Janusz Książyk

**Affiliations:** 1 Department of Pediatrics, Endocrinology, Diabetology, Metabolic Diseases and Cardiology of the Developmental Age, Pomeranian Medical University, 71-252 Szczecin, Poland; joannakastrau89@gmail.com (J.W.); klaudia.bartoszewicz@gmail.com (K.B.); mieczyslaw.walczak@pum.edu.pl (M.W.); 2 Department of Pediatrics, Nutrition and Metabolic Diseases, The Children’s Memorial Health Institute, 04-730 Warsaw, Poland; danko@ipczd.pl (M.D.); kasia.popinska@wp.pl (K.P.); msibilska@poczta.onet.pl (M.S.); janusz@ksiazyk.pl (J.K.); 3 Department of Pediatrics, Gastroenterology, Allergology and Nutrition, Medical University of Gdańsk, 80-803 Gdańsk, Poland; anna.borkowska@gumed.edu.pl

**Keywords:** parenteral nutrition, intestinal failure, metabolic bone disease, growth, children

## Abstract

Background: This cross-sectional study aimed to assess growth, body weight, and bone mineralization and to identify predictors of metabolic bone disease (MBD) in children with chronic intestinal failure (CIF) on long-term parenteral nutrition (LPN). Methods: Twenty-six children with CIF were evaluated on total parenteral nutrition (PN) for at least three years, and 60 healthy controls were assessed. Measurements included body weight, height, BMI, serum levels of 25-hydroxyvitamin D3 (25-OHD3), calcium (Ca), phosphorus (P), magnesium (Mg), and aluminum (Al), as well as urinary excretion of these elements. Dual-energy X-ray absorptiometry (DXA) and the mid-arm muscle area (MAMA) and mid-arm fat area (MAFA) of the CIF group were estimated. Results: CIF children had significantly lower height, weight, and BMI Z-scores than controls (*p* < 0.001). While the median bone mineral density (BMD) Z-score was >−2, 34.7% had L1–L4 Z-scores ≤ −2. Urinary Ca and Al excretion were higher in LPN patients, positively correlating with serum 25-OHD3 levels (r = 0.48). Lower serum Ca, P, and Mg were observed in LPN patients (*p* < 0.001), and BMC L1–L4 correlated positively with MAMA, MAFA, and BMI. Conclusions: The physical development of children who require long-term parenteral nutrition due to intestinal failure is poorer than that of healthy children. Although there are risk factors for bone mineralization disorders in children with intestinal failure, no clinical issues, such as pathological fractures, have been observed.

## 1. Introduction

Parenteral nutrition (PN) is often the only means of providing nutrition to patients with severe chronic intestinal failure (CIF), most commonly resulting from short bowel syndrome, but also to conditions such as congenital enteropathies, inflammatory bowel diseases, and gastrointestinal motility disorders [1,2,3]. Children with CIF are dependent on long-term parenteral nutrition (LPN). This therapy aims to sustain the child’s life, ensure an adequate nutritional state, and, during developmental stages, support proper growth and psychomotor development [4,5]. Individually prescribed nutritional mixtures, which depend on age, body weight, and metabolic state resulting from the underlying disease, are used. These mixtures contain macronutrients (glucose, amino acids, and lipids), electrolytes, trace elements, and vitamins [4,6].

LPN carries the risk of numerous complications. The present study focuses on complications related to growth disorders and bone metabolism. It is well established that children with CIF on LPN are at risk for reduced height and slower development compared to their healthy peers [7,8,9,10]. The first reports of metabolic bone disease (MBD) as a complication of LPN were published in 1980 and involved adult patients [11,12]. In 2010, the first study concerning pediatric patients was published [13].

MBD is defined as a condition characterized by abnormal bone mineralization, which leads to reduced bone mineral density and deterioration of bone structure [14]. The pathophysiology of MBD is multifactorial and has not yet been fully elucidated [15,16]. It may result from the underlying gastrointestinal pathology and the patient’s level of physical activity. Research has demonstrated that parenteral nutrition (PN) plays a role in developing MBD in adult and pediatric patients [17,18]. Key factors contributing to MBD include inadequate calcium (Ca) intake, an imbalance between calcium and phosphorus (P), excessive amino acid consumption, increased urinary calcium loss, and contaminants such as aluminum (Al) in the nutritional mixture [1,19,20,21,22,23,24].

Data on the prevalence of MBD in children and adolescents receiving PN vary widely. A 2022 literature review [20] reports prevalence rates ranging from 12.5% [24] to 50% [25]. According to the 2019 official position of the International Society for Clinical Densitometry (ISCD) [14], the recommended method for assessing bone health in children is dual-energy X-ray absorptiometry (DXA). The posterior–anterior (PA) spine and less head (TBLH) are recommended skeletal sites for assessing bone mineral content (BMC) and bone mineral density (BMD) in most pediatric patients. ISCD emphasizes, however, that a diagnosis of osteoporosis in children and adolescents should not rely solely on densitometric criteria. In the absence of vertebral compression fractures, osteoporosis is diagnosed based on a combination of clinically significant fracture history and an age- and sex-matched BMD Z-score ≤ −2. Nonetheless, a BMD Z-score > −2 does not exclude the possibility of skeletal fragility and an increased risk of fractures. Monitoring the development of MBD requires DXA measurement and should be part of a comprehensive assessment for children with chronic conditions that may affect bone health [14,26,27]. Additionally, meticulous monitoring of Ca, P, and vitamin D3 supply in parenteral nutrition solutions is essential, along with periodic assessment of calcium–phosphate metabolism parameters in patients undergoing such treatment [4]. Ensuring optimal bone mass accrual in chronically ill children requiring parenteral nutrition is one of the key aspects of their care.

This cross-sectional study retrospectively aimed to evaluate bone mineralization, assess growth and body weight, and identify predictors of MBD in children with CIF on LPN.

Given the limited number of studies focusing on children, this research serves as a valuable addition to the existing literature. This analysis provides new, practical insights into the long-term consequences of this therapy. Parenteral nutrition enables the delivery of nutrients while bypassing the natural processes of digestion and absorption in the gastrointestinal tract. However, this treatment method challenges the body, as it does not fully replicate natural physiological processes. Although developed per international guidelines and containing standard components such as vitamins, trace elements, and minerals, parenteral nutrition formulations are administered intravenously, which is not equivalent to a daily oral diet. Over time, this nutrition method may negatively affect a child’s growth and development and increase the risk of bone mineralization disorders.

The presented results indicate the need to deepen knowledge about the mechanisms by which parenteral nutrition affects the development of children. At the same time, they emphasize the importance of continuing research to optimize this form of treatment, which may contribute to reducing the risk of complications and improving therapeutic outcomes in the care of the youngest patients with CIF.

## 2. Materials and Methods

A cross-sectional study was conducted involving patients receiving home parenteral nutrition (HPN) from various regions of Poland. These patients were under the Department of Pediatrics, Nutrition, and Metabolic Diseases care at the Children’s Memorial Health Institute (IP-CZD) in Warsaw, the largest HPN center in Poland.

### 2.1. Study Participants

The study cohort comprised 26 children (8 females and 18 males) aged 6.3 to 18.3 years. The inclusion criterion was a minimum of 3 years of total home parenteral nutrition (HPN) due to intestinal failure (IF). Exclusion criteria included chronic corticosteroid use (patients post-liver transplantation) and bisphosphonate therapy. One female patient was excluded due to an intestinal disease genetically predisposing to short stature (microvillus inclusion disease).

Data collected from medical records included indications for PN, age at PN initiation, duration of PN, and the child’s age at the time of the DXA assessment. The characteristics of the study group are presented in Table 1.

A control group was collected to measure blood ion concentrations, urinary excretion, and to assess physical development parameters (weight, height, and BMI). It included 60 healthy children (20 females and 40 males) from northwestern Poland, matched to the study cohort by age, sex, and pubertal stage. Exclusion criteria included chronic illnesses, glucocorticoid treatment, bisphosphonates, or growth hormone.

### 2.2. Anthropometric Measurements

In all study participants (both the study and control groups), body weight and height were measured, BMI was calculated, and Tanner stage was assessed on the day of laboratory sample collection. Height was measured using a stadiometer with an accuracy of 0.1 cm, and body weight was recorded using a medical scale with an accuracy of 0.1 kg. In the retrospectively analyzed study group, data on arm muscle and fat area measurements were collected from medical records on the day of the DXA test, along with indices for muscle area of mid-arm (MAMA) and mid-arm fat area (MAFA), calculated according to standard formulas [28]. For these indices, arm circumference was measured on the dominant arm using a flexible measuring tape, recorded to an accuracy of 0.1 cm, and triceps skinfold thickness was measured using a skinfold caliper, recorded to an accuracy of 0.1 mm. Standardized values (Z-scores) were calculated for all measurements by referencing individual values against the mean and standard deviation for the Polish population [28,29]. A Z-score range of −2 to 2 was considered normal (https://ptzkd.org/standardy/, accessed on 10 November 2024). 

### 2.3. Parenteral Nutrition

On the day of laboratory sample collection, the composition of the PN mixture was specified. Each mixture contained macronutrients (glucose, amino acids, fat emulsion), electrolytes, vitamins, and trace elements. The mixtures were prepared in the hospital pharmacy, and their composition was tailored to each patient’s specific needs, considering age, body weight, and metabolic condition due to the underlying disease, per recommendations [4]. Total parenteral nutrition (TPN) was defined as the provision of at least 80% of the recommended daily calorie allowance (RDA) in line with European pediatric guidelines [6]. Patients received parenteral nutrition 7 days a week. The supply of components of the parenteral nutrition mixture in the study group is presented in Table 2.

### 2.4. Dual-Energy X-Ray Absorptiometry

The study group conducted DXA assessments retrospectively based on medical records. TBLH and spine DXA scans were performed using a fan-beam scanner (Lunar Prodigy Advance, GE Healthcare Chicago, IL, USA, enCORE software). A standard pediatric protocol covered TBLH; the lumbar region (L1–L4) was evaluated for the spine. Bone mineral content (BMC) was assessed in grams, and bone mineral density (BMD) was measured in g/cm^2^. BMD values were interpreted as Z-scores, indicating the number of standard deviations from the mean BMD values of healthy individuals of the same age group, considering sex, body weight, and height.

### 2.5. Laboratory Tests

The serum concentrations of Ca, P, magnesium (Mg), and Al and urinary excretion of these elements were measured for the study and control cohorts. Elemental analysis builds upon previous studies conducted on a larger cohort of patients, in which we investigated exposure to Al, arsenic, and cobalt in children receiving HPN. All analyses were conducted in the same laboratory. The elemental composition of serum and urine samples was determined using inductively coupled plasma mass spectrometry (ICP-MS) with a NexION 350D instrument (PerkinElmer, Norfolk, VA, USA). Urinary excretion of the analyzed elements was assessed by calculating their ratio to creatinine. The methods used for element identification have been described in detail in the previously cited articles [22,30]. In the study group, serum concentrations of albumin, alkaline phosphatase (AP), aspartate aminotransferase (AST), alanine aminotransferase (ALT), gamma-glutamyl transferase (GGT), and creatinine were also measured using standard laboratory methods. Additionally, serum levels of 25-hydroxyvitamin D3 (25-OHD3) were assessed using an automated chemiluminescent immunoassay (IDS-iSYS 25-Hydroxy Vitamin D Assay Kit, Immunodiagnostic Systems, East Boldon, UK).

### 2.6. Statistical Analysis

Descriptive statistics included the mean, median, standard deviation, minimum, maximum, and interquartile range (IQR). We assessed variable distribution using the Shapiro–Wilk test. For variables following a normal distribution, we used the *t*-test. The Mann–Whitney U test was applied to variables with a non-normal distribution or when at least one of the analyzed variables did not meet the normality assumption. Spearman’s correlation test or Pearson’s correlation test were applied according to the distribution. A *p*-value of less than 0.05 was considered statistically significant. All statistical analyses were performed using STATISTICA software (StatSoft, Inc., Tulsa, OK, USA, 2005, version 7.1).

### 2.7. Ethical Statement

The study was conducted under the guidelines of the Declaration of Helsinki and was approved by the Institutional Ethics Committee of the Pomeranian Medical University in Szczecin (KB-0012/21/2020, approval date: 9 March 2020). Informed consent to participate in the study was obtained from parents or legal guardians for children under 16. For children over 16, informed consent was obtained from the participants and their parents or legal guardians.

## 3. Results

### 3.1. Anthropometric Measurement Results

Both the physical development parameters of patients in the study group on HPN and participants in the control group were within the normal range (median Z-scores for the study group: body weight −0.9, height −0.9, BMI −0.67; median Z-scores for the control group: body weight 0.9, height 1.1, BMI 0.5). However, statistical analyses revealed significant differences between the study and control groups regarding body weight, height, BMI, and the Z-scores for these parameters.

The study group had a significantly lower median body weight (kg) and body weight Z-score compared to the control group (*p* < 0.001). In both girls and boys from the study group, body weight (kg) and body weight Z-score were significantly lower compared to the respective subgroups in the control group (for body weight: girls: *p* < 0.01, boys: *p* < 0.015; for body weight Z-score: *p* < 0.001 for both sexes).

The study group had a lower median height (cm) and height Z-score compared to the control group (*p* < 0.005 and *p* < 0.001, respectively). Analysis of these variables by sex showed that both girls and boys in the study group were significantly shorter than those in the control group (for height: girls: *p* < 0.02, boys: *p* < 0.05; for height Z-score: *p* < 0.001 for both sexes).

The study group had lower BMI and BMI Z-score values relative to chronological age than the control group (for both parameters, *p* < 0.001). BMI values were significantly lower in both girls and boys in the study group (girls: *p* < 0.02, boys: *p* < 0.001), and BMI Z-scores were significantly lower in boys (*p* < 0.001), whereas the difference in girls was not significant.

MAFA and MAMA values in the study group were within the normal range (median Z-score for MAFA was −0.2, and for MAMA −0.5), indicating no significant abnormalities in fat and muscle tissue in patients on LPN.

The results of anthropometric measurements in the study and control groups are presented in Table 3.

### 3.2. Laboratory Test Results

Table 4 displays the laboratory test results for serum and urine samples collected from the study and control groups. Serum Ca, P, and Mg levels were significantly lower in the study group than in the control group, although they remained within the normal range. Furthermore, urinary calcium excretion was higher in the study group. Aluminum concentrations in urine corresponded with serum levels, and both measurements were significantly elevated in the study group, as previously reported [22]. The median concentrations of parameters assessing liver and kidney function in patients on LPN, including AST, ALT, AP, and GGTP activity, as well as serum albumin and creatinine levels, were within the reference ranges.

### 3.3. Bone Mineral Density Results

The median 25-OHD3 concentration fell within the reference range (Table 4) [31,32]. Analyses revealed a moderate positive correlation between serum 25-OHD3 concentration and the TBLH BMD Z-score (r = 0.48). The median BMD Z-score for TBLH and L1–L4 was >−2, while a BMD Z-score of ≤−2 for L1–L4 was observed in eight patients, representing 34.7%.

Significant positive correlations were found in the study group between BMC L1–L4 and three indices: MAMA, MAFA, and BMI (r = 0.66, 0.73, and 0.75, respectively). A positive correlation (r = 0.74) was observed between Ca and Al concentrations in urine. There was no correlation between the intake of amino acids, Ca, P, and hypercalciuria. However, a positive correlation was observed between serum 25-OHD levels and urinary excretion of Ca and P (r = 0.42 and 0.41, respectively). A negative correlation was noted between TBLH BMC (g) and P intake (r = −0.5).

No significant associations were found between the duration of PN and DXA results in the conducted analyses. None of the patients had experienced bone fractures.

All test results are available in the Supplementary Materials.

## 4. Discussion

In 2022, a literature review on MBD in children on HPN was published, showing that 45% of patients with CIF have reduced bone mineral density, which correlates, among other factors, with growth disorders [20]. Data on the Polish population have been presented so far in two publications involving patients from the same center as our study group. In 2010, it was shown that the physical development of children with short bowel syndrome (SBS) was delayed compared to population norms, with growth disorders more commonly affecting boys than girls (the study included 176 patients) [10]. In 2018, it was demonstrated that SBS patients had growth deficiencies, frequent bone mineralization abnormalities, and vitamin D3 deficiency (17 patients were studied) [33].

We conducted the present study to contribute to the knowledge of bone mineralization status, growth, and nutritional status in children with SBS receiving parenteral nutrition.

In our study, physical development in the group of patients on HPN was found to be within the normal range for height, body weight, and BMI with population norms (median Z-score for height and body weight was −0.9, and for BMI, −0.67) (Table 3). However, despite these normative values, we observed significant differences between the study group and the control group of healthy children. The study group had significantly lower Z-scores for body weight and height (*p* < 0.001 for both parameters), and the differences were significant for both girls and boys (*p* < 0.001 for both sexes and both parameters). Similarly, the BMI in the study group was significantly lower than in the control group (*p* < 0.001 for BMI and BMI Z-score). Still, the difference was significant only for boys (*p* < 0.001) (Table 3).

Similar findings were reported in a study by Nader and colleagues, which included a cohort of 40 patients on HPN. In that study, body weight, height, and BMI were within normal ranges; however, height was significantly lower than the estimated target height [1]. In turn, Colomb et al. found short stature in 75% of children receiving HPN, with alternating growth acceleration and deceleration periods linked to nitrogen supply in PN. The authors attributed poor growth to a negative nitrogen balance [34]. In our study, the median amino acid supply was 1.5 mg/kg/day (range 1.2–2.0), which aligns with recommendations [4] (Table 2). No significant association was found between amino acid intake and height in our study group. In a study by Olieman et al., lower growth was observed in adult patients with SBS who had been receiving PN since early childhood, which was attributed to low energy intake and intestinal dysfunction [7]. In our patients, the energy supply from parenteral nutrition (PN) covered 80% of the recommended daily allowance (RDA), with a median of 54 kcal/kg/day (range 41–69) (Table 2), and no association with height was found. Pilcher et al. analyzed growth parameters and body composition in children with SBS on total PN. They observed a significant deficiency in height and body weight along with a high BMI; at the same time, a significantly low lean body mass index and a high-fat mass index were noted [35]. In our study group, MAFA and MAMA values were within normal ranges, with median Z-scores of −0.2 and −0.5, respectively (Table 3). This may indicate that the patients did not exhibit significant fat or muscle mass abnormalities despite lower height and body weight values. It is known that slower growth rates in children with SBS treated with LPN can be caused by various factors, including nutrient supply and intestinal inflammation [36]. Patients in our study group were adequately nourished, as confirmed by the analysis of the nutritional mixture, which was individually prepared and tailored to each patient’s current metabolic status. Basic test results assessing liver and kidney function (AST, ALT, AP, GGTP, serum albumin, and creatinine) were within normal limits (Table 4). Normal values for height, body weight, and BMI relative to population norms further indicate an adequate nutritional status. However, a comparison with the control group suggests poorer physical development in patients on LPN. We believe this may be related to the chronic nature of their condition, involving frequent hospitalizations, limited physical activity, and increased susceptibility to infections. The lower height and body weight in our study group are most likely due to severe CIF and multiple complications related to the underlying disease, rather than the effect of PN. This hypothesis is supported by findings from Nader’s study, in which no growth slowdown was observed within 6 months after discontinuing PN [2]. In turn, Pichler and colleagues demonstrated that the main factor limiting growth was intestinal disease rather than PN; children with enteropathy and inflammatory conditions of the intestinal mucosa exhibited the lowest height [36]. We were unable to conduct such an analysis in our patients due to the small sample size and considerable diversity in indications for HPN.

Bone mineralization was assessed in all patients after at least two years of HPN, as this period allows for demonstrating the impact of PN on bone health [1]. According to the ISCD definition, osteoporosis was not diagnosed in any patient, as no bone fractures were observed, and a Z-score ≤ −2 was considered low [14]. The median BMD Z-score for TBLH and L1–L4 was >−2. However, analysis showed that the BMD Z-score for L1–L4 was ≤−2 in 34.7% of the patients. Similar results were observed in previous studies, with Nader et al. reporting 30% [1] and Pichler et al. reporting 31% [36].

We analyzed predictors of bone mass in our cohort. The results of our study showed a significant increase in urinary excretion of Ca and Al in patients on LPN compared to the control group (*p* < 0.002 and *p* < 0.012, respectively; see Table 4). A strong positive correlation between urinary Ca and Al concentrations suggests that Al may contribute to increased Ca excretion, potentially being one of the mechanisms leading to reduced bone mineralization and weakening of bone structure. Aluminum administered intravenously as a contaminant in parenteral nutrition mixtures interacts with Ca and exhibits a strong affinity for phosphates, affecting mineral metabolism, particularly during prolonged exposure to its sources [22,35,37]. Aluminum accumulation in bone tissue impacts osteoblast function, disrupting bone formation [38]. During bone mineralization, Al replaces Ca in unmineralized type I collagen. Additionally, it accumulates in the parathyroid glands, impairing parathyroid hormone (PTH) secretion. Aluminum also inhibits the absorption of intravenously administered Ca, which may result in hypercalciuria. These mechanisms collectively contribute to the development of osteomalacia [19]. In our study, we did not find a relationship between Al concentrations in serum and urine and BMC or BMD values, which aligns with the findings of Appleman et al. [39]. It is known that increased urinary Ca loss plays a role in the development of MBD [37]. Among the factors influencing hypercalciuria in patients receiving parenteral nutrition, aside from the previously mentioned Al, excessive intake of amino acids and Ca and phosphate deficiency are noted [25,40,41,42]. Larchet et al. suggest, however, that hypercalciuria in patients receiving parenteral nutrition may be caused by excessive vitamin D3 intake [25]. We analyzed the supply of all components of the nutritional mixture in our study group (Table 2). We found no association between the intake of amino acids, Ca, P, and hypercalciuria. However, a moderate positive correlation was observed between serum 25-OHD levels and urinary excretion of Ca and P, suggesting that fluctuations in vitamin D levels may significantly impact calcium–phosphorus homeostasis. Excessive vitamin D levels lead to increased intestinal Ca absorption, which, in turn, places greater strain on the kidneys and exacerbates hypercalciuria.

At the same time, a moderate positive correlation was found between serum 25-OHD3 levels and TBLH BMD Z-score, indicating a beneficial effect of vitamin D on bone mineral density.

In turn, the negative correlation between TBLH BMC (g) and P intake suggests a harmful effect of excessive P intake on bone mineralization. This phenomenon can be explained by a physiological mechanism in which excess dietary P leads to increased PTH secretion, which promotes bone resorption and thus may reduce bone mineral density. Studies from Takeda et al. [43] and Calvo and Park [44] support this. Additionally, excessive P intake may disrupt Ca metabolism, which plays a crucial role in proper bone mineralization [45].

The observed lower median serum concentrations of Ca, P, and Mg in children on LPN, compared to the control group, while remaining within the normal range, suggest subclinical deficiencies that may, over time, lead to weakened bone structure and an increased risk of fractures. Aluminum overload, found in both groups with higher values in the control group, requires further analysis regarding aluminum sources and their impact on bone health.

Our cohort analysis of bone mass predictors demonstrated statistically significant positive correlations between BMC L1–L4 and three indicators: MAMA, MAFA, and BMI. This indicates that the anthropometric parameters examined positively correlate with bone mineral content.

Several studies have analyzed the impact of PN duration on the development of MBD. A negative correlation between PN duration and BMD was reported in studies by Neelis et al. [21], Demehri et al. [46], and Mutanen et al. [18]. In contrast, Poinsot et al. [15] described significantly reduced MBD risk with prolonged PN duration. In our cohort, with a median PN duration of nearly 9 years, no significant correlations were found between PN duration and BMD or BMC indicators, consistent with the findings of Diamanti et al. [13]. Similar observations were reported by Nader et al., showing that long-term PN (over 10 years) does not increase the risk of developing MBD [1].

## 5. Conclusions

- The physical development of children who require long-term parenteral nutrition due to intestinal failure is poorer compared to that of healthy children;
- Although there are risk factors for bone mineralization disorders in children with intestinal failure, no clinical issues, such as pathological fractures, have been observed.

## Figures and Tables

**Table 1:** Characteristics of the study group.

Parameter	Min–Max	Median
Age of onset of PN (months)	0.13–135.4	3.07
The duration of PN until the day of DXA scan (months)	36–195	104
Age on the day of the DXA examination (months)	52–195	128.5
Age on the day of serum and urine collection (months)	76.47–219	151.1
**Indication for PN**		**Number of Patients**
Mesenteric torsion		2
Congenital malformation of the mesentery		2
Torsion of the small intestine		5
Intestinal atresia		1
Hirschprung’s disease		5
Absorption disorders		2
Neurogenic bowel		2
Necrotizing enterocolitis		3
Incomplete intestinal diversion		1
Pagoda syndrome		1
Expectoration		2

PN—parenteral nutrition, DXA—dual-energy X-ray absorptiometry.

**Table 2:** Supply of PN mixture components in the study group.

Components of a Parenteral Nutrition Mixture	Supply Median (Min–Max)	IQR
Carbohydrates (g/kg/day)	7.4 (6.0–11.3)	7.4
Amino acids (g/kg/day)	1.5 (1.2–2.0)	0.1
Lipids (g/kg/day)	1.5 (1.2–2)	0.6
Calcium (mg/kg/day)	0.2 (0.1–0.45)	0.1
Phosphates (mg/kg/day)	0.1 (0.1–0.3)	0.095
Magnesium (mg/kg/day)	0.1 (0.1–0.44)	0.05
Trace elements (Peditrace^®^, Fresenius Kabi) (mL/kg/day)	0.5 (0.25–1.0)	0.2
Vitamin D3 (IU/day)	200 (200–400)	200
Volume of PN (mL/kg/day)	51 (47–64)	6.5
Calories (kcal/kg/day)	54 (41–69)	11.75

IQR—interquartile range.

**Table 3:** Results of anthropometric measurements in the study and control groups by sex.

	Study Group			Control Group			*p*-Value
	Group Size: -Whole Group -Girls -Boys	Median (Min–Max): -Whole Group -Girls -Boys	IQR: -Whole Group -Girls -Boys	Group Size: -Whole Group -Girls -Boys	Median (Min–Max): -Whole Group -Girls -Boys	IQR: -Whole Group -Girls -Boys	
Body weight (kg)	26 8 18	33.6 (22.8–60) 34 (26.7–49) 29.3 (14.9–59.4)	12 15.7 12.45	60 20 40	49.5 (19.7–82) 46.5 (34.2–62.4) 48.2 (19.7–82.1)	25.4 16.2 33.1	<0.001 <0.01 <0.015
Body weight Z-score (SD)	26 8 18	−0.9 (−3.5–1) −1.03 (−1.45–0.05) −0.92 (−2.3–0.68)	0.5 0.38 0.89	60 20 40	−0.1 (−1.07–2) −0.015 (−1.07–0.84) −0.26 (−0.98–1.57)	0.9 0.71 0.78	<0.001 <0.001 <0.001
Body height	26 8 18	142.5 (122.1–169) 137.1 (125–159) 139 (101.1–166)	24.3 29.4 26.45	60 20 40	158.7 (112–184) 157.7 (140–175.3) 158.9 (112–184.2)	25.4 16.6 45.4	<0.005 <0.02 <0.05
Body height Z-score (SD)	26 8 18	−0.9 (−7.6–1) −1.3 (−2.7–−0.56) −0.87 (−4.7–0.78)	0.5 1.39 1.54	60 20 40	0.1 (−1.4–2) 0.44 (−1.2–1.86) −0.28 (−1.4–1)	1.1 0.83 0.75	<0.001 <0.001 <0.001
BMI kg/m^2^	26 8 18	16.4 (14.3–23.4) 17.5 (16.01–19.3) 15.57 (14.3–23.5)	2.59 1.84 1.89	60 20 40	19.2 (15.7–25) 18.91 (16.46–22.4) 19.68 (15.7–25.06)	3.99 2.94 4.44	<0.001 <0.02 <0.001
BMI to calendar age Z-score (SD)	26 8 18	−0.67 (−2.45–0.83) −0.42 (−1.3–0.03) −0.74 (−2.45–0.83)	0.9 0.79 1.02	60 20 40	−0.12 (−0.9–1.37) −0.065 (−0.9–0.74) −0.07 (−0.93–1.37)	0.5 0.59 0.45	<0.001 NS <0.001
MAMA to age growth Z-score (SD)	26	−0.5 (−1.48–2)	1	-	-	-	-
MAFA to age growth Z-score (SD)	26	−0.2 (−2.11–2)	0.7	-	-	-	-

IQR—interquartile range, NS—not significant, BMI—body mass index, MAMA—muscle area of mid-arm, MAFA—mid-arm fat area.

**Table 4:** The laboratory test results for serum and urine samples from the study and control groups.

	Study Group			Control Group			
Serum	Group Size	Median (Min–Max)	IQR	Group Size	Median (Min–Max)	IQR	*p*-Value
Calcium (mmol/L)	26	2.39 (1.84–2.93)	0.092	60	2.53 (2.36–3.11)	0.164	<0.001
Phosphorus (mmol/L)	26	1.38 (0.7–2.1)	0.4	60	1.7 (1.25–2.15)	0.276	<0.001
Magnesium (mmol/L)	26	0.78 (0.57–0.98)	0.118	60	0.89 (0.78–1.06)	0.083	<0.001
Aluminum (μg/L)	26	7.9 (3.3–21)	5.3	60	56.3 (29.36–414)	23.2	<0.001
25-OHD3 (ng/mL)	26	26.6 (16.7–49.7)	9.9	-	-	-	-
Albumin (g/l)	26	42.7 (36.3–48)	4.9	-	-	-	-
AST (U/L)	26	31 (15–113)	12.25	-	-	-	-
ALT (U/L)	26	31.5 (17–153)	17.15	-	-	-	-
GGTP (U/L)	26	20 (9–79)	11	-	-	-	-
Creatinine (mg/dl)	26	0.4 (0.28–1)	0.6	-	-	-	-
AP (U/L)	26	290.5 (143–745)	81	-	-	-	-
**Urine**							
Calcium (μg/g creatinine)	26	674,290 (1866.46–18,086,634)	23,81,366.1	60	412,800.1 (18,492.24–2,540,402)	575,843	<0.002
Phosphorus (μg/g creatinine)	26	80 (0.18–476)	137.6	60	133.4 (5.76–747)	143.2	NS
Magnesium (μg/g creatinine)	26	131,108.1 (70.13–1,225,064)	386,999	60	85,133.8 (9772.95–415,421)	71,606	NS
Aluminum (μg/g creatinine)	26	41.6 (0.84–2762)	141.5	60	8.7 (1.6–69)	10.2	<0.012

IQR—interquartile range, NS—not significant.

## Data Availability

The data presented in this study are available upon request from the corresponding author.

## Supplementary Materials

The following supporting information can be downloaded at https://www.mdpi.com/article/10.3390/nu17040611/s1: Table S1: Database study group and control group.
